# Food and Feeding

**Published:** 1904-12-24

**Authors:** 


### Food and Feeding.

Few things can be more curious than the change which has been passing over the public mind, in the course of the last few years, with reference to food and feeding. Much has been made from time to time of the tendency to glorify intoxication which rollicks through the pages of Dickens, while it has to some extent escaped notice that his types of character are, as a rule, quite as much given to over-eating as to over-drinking; and that the meals at Manor Farm are as much in evidence as the beverages. Underlying the whole fabric of his fiction is an assured belief that to eat a great deal of food is an action not only meritorious in itself, but certain to be rewarded by good health, high moral tone, and enhanced physical vigour; and it is not too much to say that these were the prevailing convictions of the time in which he lived. The late Sir Henry Thompson, in the interesting volume upon Food and Feeding, which has now passed through many editions, was among the first to insist forcibly upon the fact that the amount of food consumed should be much diminished in advancing life; and Professor Clifford Allbutt has lately written to the *Times*, on his return from a tour in America, to say that wholly new ideas about diet are beginning to prevail there, and that American men of science have obtained demonstration of the fact that all food in excess of the actual requirements of the body is a source of weakness instead of a source of strength; nervous force which might be better employed being consumed in its partial digestion and in its ultimate removal from the system. He tells us that this is especially true of nitrogenous material, while hydrocarbons can be burnt off in respiration with comparative facility. It would almost seem as if Nature, in attaching pleasure to the consumption of food, had carried her precautions against starvation to excess, and had ignored the risks incidental to repletion. An inquest was held a week or two since upon an omnibus driver who died suddenly after an enormous meal of pork chops, and from no other discoverable cause than gastric distension by food and flatulence which arrested the action of a feeble heart.

A considerable amount of evidence has lately been brought forward from one source and another tending with remarkable uniformity to show that the human body can be maintained in full vigour and activity upon a much smaller amount of food than is usually consumed, and the question is one which calls for the serious attention of physicians and of physiologists. It has unfortunately been very much left, up to the present time, in the hands of minorities, who may, without gross exaggeration be described as faddists, and hence it has not received the consideration due to its manifest importance. A non-medical contemporary has lately described a "vegetarian Christmas dinner," held at a restaurant in Furnival Street, as "a strange and horrible orgy," apparently on no other ground than that no meat entered into its composition, and predicted that its chief effect upon the guests would be "an exceptional display of vigour in attacking chops and steaks" on the following day. We can feel for the distresses of a writer who is compelled to furnish his daily tale of "copy" without assistance from the very binding form of straw which is commonly denominated knowledge, but where was the editor? The question of the kind of food consumed is, however, a different one from the question of quantity; and our present object is to suggest that the majority of people eat a great deal too much, and fall short of their highest attainable standard of mental and bodily activity in consequence. There must obviously be a limit to the needs of the body, and when the supply exceeds this limit it must obviously be removed or disposed of in some way. It can scarcely be doubted that the American view quoted by Professor Clifford Allbutt is essentially correct, and that the disposal of the superfluity is a tax upon the organism by which it is effected. It is at least not doubtful that the period of gorging, of which the Dickens era was perhaps the termination, was also a period of premature decease among the well-to-do, or that a great increase of longevity has been a characteristic of more recent times. Fifty years ago an octogenarian able to take part in the activities of life was at least regarded as a remarkable exception to the common rule; to-day, this position could hardly be sustained. Apart from and beyond the needs of individuals, the question is one not destitute of national importance. It has a serious bearing upon the cost of living; and in easily conceivable circumstances it might have a still more serious bearing upon the power of national resistance in time of war. The waste which is trivial in the case of the individual becomes enormous when it is practised by a great community; and if, as seems highly probable, this waste applies not only to foodstuffs, but also to the bodily health and the intellectual capacity of the people, it is one that should be checked, as far as possible, by all the influences of education and of public opinion.

